# Development, implementation and evaluation of a clinical research engagement and leadership capacity building program in a large Australian health care service

**DOI:** 10.1186/s12909-016-0525-4

**Published:** 2016-01-14

**Authors:** Marie L. Misso, Dragan Ilic, Terry P. Haines, Alison M. Hutchinson, Christine E. East, Helena J. Teede

**Affiliations:** Monash Centre for Health Research and Implementation, School Public Health and Preventative Medicine, Monash University, MHRP, 43-51 Kanooka Grove, Clayton, VIC 3168 Australia; Department of Epidemiology & Preventive Medicine, School of Public Health & Preventive Medicine, Monash University, Level 6, The Alfred Centre, 99 Commercial Rd, Melbourne, VIC 3004 Australia; Physiotherapy Department, School of Primary Health Care, Monash University, Clayton, 3168 VIC Australia; Allied Health Research Unit, Monash Health, Kingston Centre, Kingston Rd, Cheltenham, VIC 3192 Australia; Deakin University and Monash Health Partnership, Centre for Nursing Research, Monash Medical Centre, 246 Clayton Rd, Clayton, VIC 3168 Australia; Monash Women’s Maternity Services, Monash Health and School of Nursing and Midwifery, Monash University, Monash Medical Centre, 246 Clayton Rd, Clayton, VIC 3168 Australia

**Keywords:** Research capacity building, Clinical research, Medical education, Leadership, Evidence-based practice

## Abstract

**Background:**

Health professionals need to be integrated more effectively in clinical research to ensure that research addresses clinical needs and provides practical solutions at the coal face of care. In light of limited evidence on how best to achieve this, evaluation of strategies to introduce, adapt and sustain evidence-based practices across different populations and settings is required. This project aims to address this gap through the co-design, development, implementation, evaluation, refinement and ultimately scale-up of a clinical research engagement and leadership capacity building program in a clinical setting with little to no co-ordinated approach to clinical research engagement and education.

**Methods/Design:**

The protocol is based on principles of research capacity building and on a six-step framework, which have previously led to successful implementation and long-term sustainability. A mixed methods study design will be used. Methods will include: (1) a review of the literature about strategies that engage health professionals in research through capacity building and/or education in research methods; (2) a review of existing local research education and support elements; (3) a needs assessment in the local clinical setting, including an online cross-sectional survey and semi-structured interviews; (4) co-design and development of an educational and support program; (5) implementation of the program in the clinical environment; and (6) pre- and post-implementation evaluation and ultimately program scale-up. The evaluation focuses on research activity and knowledge, attitudes and preferences about clinical research, evidence-based practice and leadership and post implementation, about their satisfaction with the program. The investigators will evaluate the feasibility and effect of the program according to capacity building measures and will revise where appropriate prior to scale-up.

**Discussion:**

It is anticipated that this clinical research engagement and leadership capacity building program will enable and enhance clinically relevant research to be led and conducted by health professionals in the health setting. This approach will also encourage identification of areas of clinical uncertainty and need that can be addressed through clinical research within the health setting.

## Background

The benefits to patient care and service delivery can be greatly enhanced with research that is led by the health professionals who will use it, making it relevant to the health care setting. The benefits of health research designed to deliver relevant healthcare improvements have the potential to span the patient experience, improvements in healthcare outcomes and to facilitate efficient use of resources [1]. The McKeon review of Health and Medical Research in Australia identified that researchers need to engage more directly with clinicians and other stakeholders to ensure that research addresses key clinical needs and gaps and provides practical and implementable solutions [2]. The review recommended that health and medical research be fundamentally embedded in the health system. It also recommended involving the healthcare delivery workforce in research to drive a continuous improvement mindset, where research is carried out in a purposeful manner, valued and rewarded; outcomes and impacts–beneficial or detrimental–are tracked and evaluated; and a feedback system is in place to inform research gaps [2].

The literature about engaging health professionals in conducting and leading research is still evolving with ongoing initiatives seeing positive progress. A program of research funded by the Canadian Institutes of Health Research describes a collaborative model that aims to involve health professionals in the design of research, with a number of health practitioners named as co-investigators, to ensure that the research is designed to be relevant to the needs of practice [3–5]. This approach instilled a sense of shared ownership of the research findings among the researchers and health practitioners [4].

In the UK, the National Institute for Health Research Collaborations for Leadership in Applied Health Research and Care (CLAHRCs) are collaborative partnerships between universities and healthcare organisations that facilitate capacity building and engagement of healthcare organisations to conduct and apply high quality clinical research focused on the needs of patients [6–10].

In Australia, the Queensland Physiotherapy Rehabilitation Network promotes professional-led research where health professionals from rehabilitation units and researchers work together to generate research findings relevant to the health professional’s practice [11]. Health practitioner engagement in clinical research increased among allied health and oral health practitioners in a health service in Queensland, Australia through creation of research positions, awarding of research grants for clinically based research questions, and establishment of leadership, support and governance [12].

High quality, effective clinical research inclusive of clinical trials and health care improvement or health services research requires capacity building of the individual health professionals and the organisation. The health system itself needs to provide a sustainable and supportive environment for health professional-led research. These concepts can be guided and measured using the research capacity building framework proposed by Cooke [13, 14]. However, empirical evidence evaluating interventions focussed on clinical research capacity building among health professionals within a health system is still required.

Grol [15] suggests that a one-off educational activity is seldom effective to introduce such interventions, whereas a well-planned, integrated approach that accounts for the complex reality of clinical practice is superior. Effective elements are multifaceted and include defining a well-justified and attainable proposal; engaging with health professionals at all stages; and incorporating a mix of strategies with continuous monitoring of progress, feedback, and adaptation of strategies as needed [15]. It is envisaged that embedding a new strategy within day-to-day activities has the potential to encourage users to adopt it without viewing it as extra work and strategies tailored to the needs of the health professionals are more likely to be embraced [16].

Cook [14] developed a framework for planning and measuring progress of a research capacity building program that can be applied at an individual, team and organisational level within a particular context. It is based on an analysis of the literature and includes the following principles: “develop skills and confidence, support linkages and partnerships, ensure the research is ‘close to practice’ , develop appropriate dissemination, invest in infrastructure, and build elements of sustainability and continuity”. This framework was used to increase the research capability of health professionals in healthcare settings in the UK and was found to be effective for health professionals that were in a supportive organisation, where they could be freed from clinical duties; and where there were colleagues with research experience [17].

Using an action research approach and research capacity building outcome framework, we will draw upon the cited strategies to develop, implement and evaluate a clinical research engagement and leadership capacity building program. This program will be implemented in a health setting where current education strategies focus on clinical care training, with little to no coordinated approach to provision of education in evidence-based practice, research methodology or leadership in the clinical context. We hypothesise that the program will enable, enhance and support a sustainable culture of health professional-led research and evidence-based practice across all levels of the healthcare setting.

### Scenario of current practice enhanced by research-enabled health professionals

**Table:** 

Activity	Expertise	Example
Identification of problem in healthcare setting: poor outcomes for patients with a specific clinical condition; or inefficient health care practices.	Clinical knowledge Stakeholder engagement Diagnostic research	ICU patients with vascular catheters for renal replacement therapy are not allowed to mobilize for fear of safety or equipment disruption despite evidence that mobilization improves health outcomes.
Identify evidence based recommendations for addressing poor outcomes.	Evidence-based practice	Recommendations were sought by clinical team but not identified.
If no clear evidence based recommendations, start defining them.	Evidence-based practice Stakeholder engagement Clinical knowledge	Local recommendations and procedures developed.
Review the evidence for interventions to address poor outcomes.	Evidence-based practice	No specific evidence found investigating whether it is safe for the patient and equipment to mobilize these patients.
If no evidence, start generating the evidence in local healthcare setting as a healthcare improvement research activity to address poor outcomes.	Lead and engage clinical team and health care setting. Prioritisation of clinical research (allocate time and resources away from clinical tasks) Specific skill set in conducting the research to determine the best way to address the poor outcomes.	Trial designed by clinical team in partnership with researchers to address this issue. Ethics approval gained and trial commenced on site.
Use in-house research findings to improve outcomes for patients and establish individuals, team and health care setting as leaders in clinical research for specific condition.	Clinical knowledge AND understanding of value of rigorous research.	Trial found no safety concerns for patients or equipment. Research published [18]. Local procedures changed in light of findings.

## Methods

### Setting

Monash Health is one of Australia’s largest health services and provides health care across the entire lifespan – from pre-birth, newborn babies and children, to the aged, their families and carers. Services are provided through more than 40 locations across south-east Melbourne, Victoria, including: high acuity teaching and research hospitals and community hospitals; mental health care through hospital, community and outreach services; chronic disease management; primary care through community health centres; prevention and early intervention. Monash Health is a clinical partner in research that is advancing knowledge and clinical care as a core member of Monash Partners, a recently accredited Academic Health Sciences Centre. Monash Partners includes seven independent providers of health services, health research and health education in the South East of Melbourne, Australia; and is recognised by the Commonwealth. These include Monash Health, Alfred Health, Cabrini Health, Epworth Health, Monash University, and the research institutes (Hudson Institute, The Burnet and Baker IDI). Within Monash Partners, one of the largest Academic Health Sciences Centres in the world, the Monash Centre for Health Research and Implementation (MCHRI) is designed to deliver rigorous research findings, clinical research and leadership training to build capacity in healthcare improvement and to support clinical research and implementation at Monash Health. This provides an ideal environment to co-design, develop, implement, evaluate, refine and ultimately scale-up education and support programs to drive direct improvement in health care outcomes.

### Design

A mixed methods study design utilising both quantitative and qualitative research methodology will be utilised. An action research approach will be facilitated through the partnership between MCHRI and Monash Health. Ethics approval has been obtained from the Monash Health Human Research Ethics Committee.

**Fig. 1:**
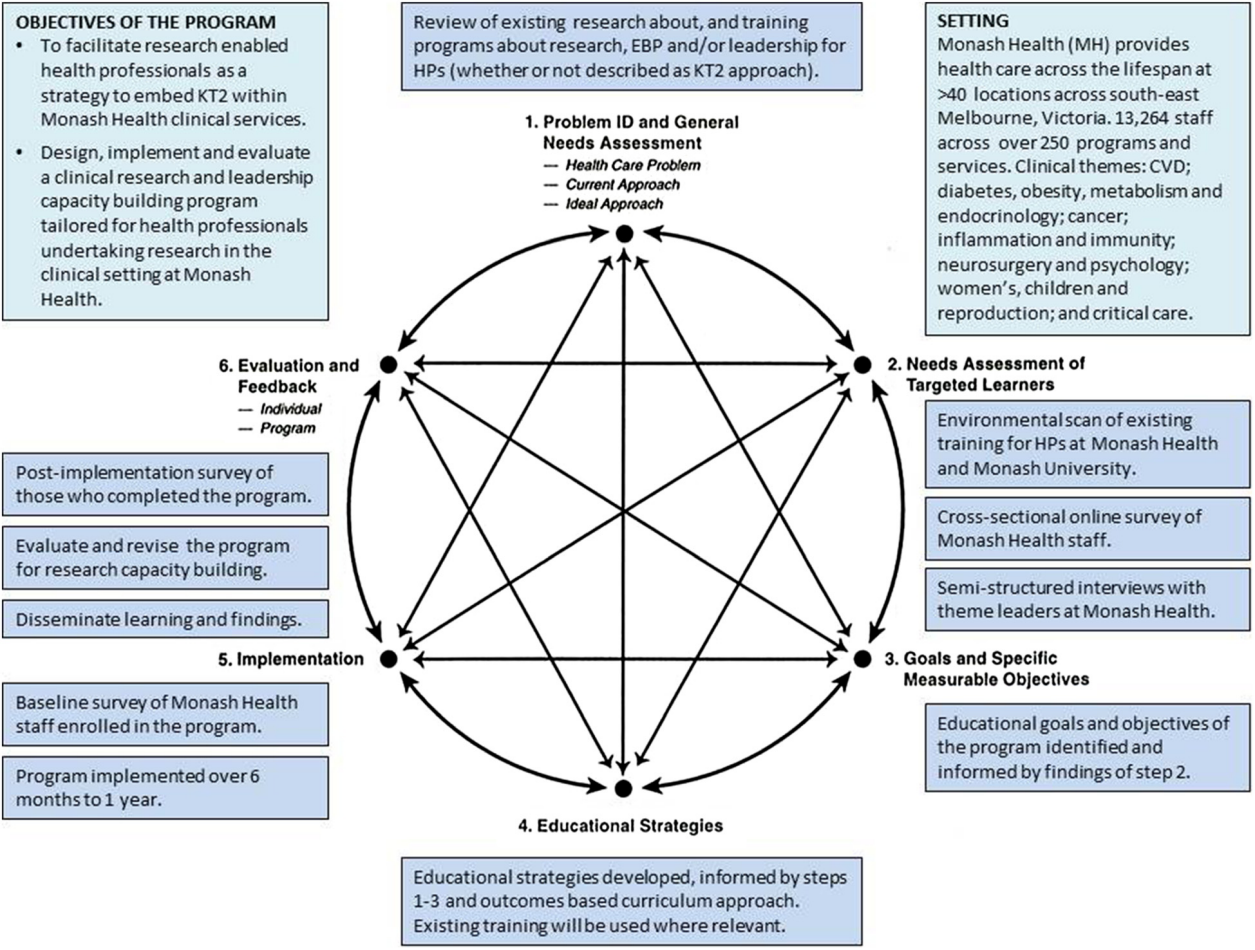
Outline of project methods underpinned by the six-step approach to curriculum development for medical education, adapted from Kern 1998 [19]. KT2, knowledge translation 2; EBP, evidence-based practice; HPs, health professionals; MH, Monash Health; CVD, cardiovascular disease

The principles of planning change and measuring progress of research capacity building [14] and the six-step approach described by Kern [19] for development of medical education curriculum will be employed in order to guide the development, implementation and evaluation of the proposed clinical research engagement and leadership capacity building program. The six-step curriculum development model is well-established for the design of medical education and has been shown to lead to long-term sustainability; and the approach advocates linking of education to health care needs (Fig. 1) [19].

### Step 1 - Problem identification and general needs assessment to inform development of the training program

A systematic review of literature about the content and effectiveness of strategies or programs designed to build capacity and/or engagement for health professional-led research will be conducted. Electronic databases (to identify published literature), such as MEDLINE, EMBASE, All EBM and Health Systems Evidence; and Google (to identify grey literature) will be systematically searched combining the following terms that will be used as subject headings and text words (including variations such as those related to spelling, tense or synonyms and related terms) as appropriate: clinical research; research design; evidence based practice; healthcare improvement; leadership; medical education; capacity building.

Articles will be included if they describe the content of, or effectiveness of, education or support delivered with the aim of building capacity or educating health professionals about two or more of: evidence-based practice; clinical research methods; clinical research leadership; or healthcare improvement. Articles describing undergraduate curricula will be excluded and postgraduate curricula will only be included if their target audience is health professionals.

Articles published in journals, describing studies or syntheses of studies that meet the selection criteria, will be critically appraised using templates designed according to study design; and extracted for key themes associated with research capacity building strategies; content of, and effectiveness of education programs/elements and support. Grey literature such as training program websites or reports will be reviewed for key themes. Key themes will be compiled using an inductive approach and the resulting coding framework will inform further stages of the project. The findings of the systematic review will inform the development of the strategies and content, where aligned with local needs assessment, of the clinical research engagement and leadership capacity building program.

### Step 2 - Local needs assessment of targeted learners in the training program

A. A document review of existing support and education elements within Monash Health and Monash University for health professionals about evidence-based practice principles; clinical research methods; clinical research leadership; or healthcare improvement will be conducted. The review will document each organisation’s websites and any informal liaison with key informants involved in the delivery of support and education to health professionals at each organisation. Findings will be tabulated.
B. The needs and preferences of health professionals within Monash Health will be explored to inform the content and delivery methods of the clinical research engagement and leadership capacity building program, through an online survey and semi-structured interviews. The questions in the survey and semi-structured interviews will be framed in constructs according to the theory of planned behaviour [20].

#### Cross-sectional online survey

Monash Health staff will be invited by email to participate in an online survey [21]. The link to the survey will be provided in the same emailed invitation. Participant consent will be implied upon completion of the survey. The survey will collect data using Likert scales and open ended fields about their current practice, knowledge, confidence, attitudes, perceived behavioural control and barriers in relation to using research - evidence-based practice (EBP), conducting clinical research and clinical research leadership in the participant’s role at Monash Health. The survey has been developed by the investigators, based on a questionnaire developed by Jette et al., designed to explore respondents’ attitudes and beliefs about EBP; interest in and motivation to engage in EBP; educational background and knowledge and skills related to accessing and interpreting information; level of attention to and use of the literature; access to and availability of information to promote EBP; and their perceived barriers to using evidence in practice [22]. This tool was also used to construct questions relevant to conduct of clinical research. The Central Michigan University Leadership Competency Assessment [23] was used to inform the clinical research leadership component of the survey and the Massachusetts Institute of Technology Training Delivery Methods Survey [24] was used to inform the education delivery component. Strategies used to maximise participation in the survey include: (1) The invitation to participate in the survey will be sent through the email of relevant senior clinical leaders and Monash Health executive staff to promote the importance of the project; (2) survey participants will be invited to submit an email entry into a draw to win cinema tickets.

#### Semi-structured interviews

Key informants for developing the program will be identified by their role and expertise in their clinical area; as well as their role as a representative for their team and the potential needs with respect to conducting and using research. This step of the needs assessment will also facilitate engagement with potential senior clinical research leaders and champions within Monash Health who may have the ability to prioritise and facilitate their team’s engagement with the program. Knowledge of existing education elements or capacity to provide education will also be very valuable. Clinical theme and research program leaders within Monash Health (Cardiovascular disease; Diabetes, obesity, metabolism and endocrinology; Cancer; Infection and immunity; Neurosurgery and psychology; Women’s, children and reproduction; Health services, Public health, Innovation; Research directorate and services; Allied health; Quality and innovation; and the Centre for Clinical Effectiveness) will be invited by email to participate in a semi-structured interview to inform the content and delivery of the program. A participant information and consent form (approved by the Monash Health Human Research Ethics Committee) will be provided and explained further verbally before obtaining written informed consent and proceeding with the interview. The interview questions will follow the headings of the online survey with prompts to gather further in-depth information. All participants will be informed that their responses to the interview will be transcribed and unable to be identified. Participants will also be informed that their responses may be used for educational research and development of the program. Where possible, interviews will be conducted by the same facilitator and will be conducted either by phone or face-to-face, according to the preference of the participant. Interviews will be recorded via digital recorder and transcribed. Transcripts will be de-identified for analysis. Thematic analysis [25] of responses will be performed by two independent researchers and a coding framework will be developed.

### Step 3 - Goals and specific measurable objectives

Findings from steps 1 and 2, incorporating the systematic literature review, document review of existing education elements and the local needs assessment, will be examined and translated into education goals and learning objectives for the education component of the program. A Delphi approach [26] of repeated online surveys will be implemented with the investigators to determine final education goals and learning objectives.

The theory of symbiosis [27] between the curriculum, the health service and communities in which the health professionals will practice; and guidelines for inter-professional education and collaborative practice [28] will inform this step in order to ensure that the education elements contain the required learning material to facilitate use of and engagement with clinical research and adoption of evidence-based healthcare improvement practices within Monash Health.

### Step 4 – Engagement strategies: education and support

**Fig. 2:**
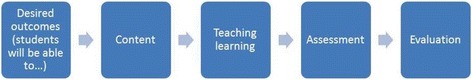
Steps for developing training program content, adapted from Prideux 2003 [29]
B. Support strategies will be informed by steps 1–3 and will particularly draw on findings about barriers and perceived behavioural control to address suitability and sustainability of the program.

A. Educational strategies will be informed by steps 1–3. An outcomes (goals and objectives) based curriculum approach described by Prideaux [29] will be used (Fig. 2). The content of the program will be developed based on a starting point, in this case, the knowledge gaps that need to be addressed (identified at step 2 A and B), with the outcome being a closure of that gap in knowledge, through education and support.

### Step 5 – Dissemination and implementation

The program, once developed, will be implemented at Monash Health with education elements available to all Monash Health staff. Wherever possible, existing education elements at Monash Health and Monash University (and where appropriate external online resources), identified in step 2A, will be used in the program. Education modules will be developed with content experts. The format and delivery of education modules (e.g. face-to-face, online) will be informed by the needs assessment. It is envisaged that the program will be implemented over 6 months to 1 year. Depending on the findings at step 2B, the program may consist of online and face-to-face education modules with accompanying assessment tasks. Dissemination will be conducted through internal Monash Health communication channels.

#### Baseline/pre-implementation survey

Before implementation of the program, a baseline online survey will be conducted among program participants to determine current practice and behaviour, knowledge and attitudes about the education element(s). The survey will be developed by the investigators based on the education modules that will be included in the program; and a survey approach similar to that used for the needs assessment survey will be employed. A unique identifier (year of birth and first three letters of mother’s maiden name) will be incorporated into the survey in order to allow data linkage between the pre- and post-implementation surveys. Data will be collected using Survey Methods (www.surveymethods.com).

### Step 6 – Evaluation and feedback

At the completion of each education module, participants will be asked to complete a short evaluation survey to determine suitability and relevance of the module. At the completion of the program, participants will be invited to complete a more comprehensive evaluation survey, with the same questions as that of the baseline survey to enable direct comparison between pre- and post-implementation of the program. After the first round of the program, the investigators will evaluate the feasibility, suitability and effect of the program, in terms of knowledge, practice of learned skills and attitudes, based on survey data. The program will be revised accordingly. The research capacity building criteria proposed by Cook [14], which will inform the outcomes measured at the individual, team, organisational and external engagement level to determine the effect of the program to build research capacity [14, 30], will be captured by the surveys in the short and longer term. Additionally, established linkages, partnerships and collaborations; and research conducted at Monash Health and its dissemination and impact will be conducted through a search of Scopus to identify research published with a Monash Health by-line pre-implementation compared to at 1 and 2 years post-implementation of the program. Research projects and funding registered with the Monash Health Research Governance office will also be documented pre- and post-implementation of the program.

### Scale-up

The program will then be offered across Monash Partners and affiliated organisations. Translation to the other three Academic Health Sciences Centres is also planned.

### Data analysis

Quantitative and qualitative data will be collected and analysed. The needs assessment survey, baseline and post-implementation surveys will use scales (including a 5 point Likert scale) and open ended fields. Descriptive statistics will be used to analyse quantitative survey data and comparisons between pre- and post-intervention surveys will be conducted using relevant regression analysis techniques (linear regression for continuous outcomes, ordered logit regression for ordinal/Likert-scaled items). Statistical analyses will be performed using Stata [31]. Qualitative thematic analysis of open ended data and interviews will be performed with the assistance of NVivo [32]. Transcripts from interviews will be de-identified for thematic analysis, performed by two independent researchers and a coding framework will be developed.

## Discussion

Greater empirical evidence evaluating strategies that focus on engagement and building capacity in clinical, health care improvement and health services research among health professionals is required to reduce the evidence-practice gap across different populations and settings. Evidence suggests that there is a sense of shared ownership of emerging findings among researchers and health professionals when health professionals are involved in the design of the research [3–5]. This approach also ensures that the research addresses the needs of clinical practice. An integrated approach that accounts for the complex reality of clinical practice and incorporates a practical and well-informed proposal for change through engaging with health professionals at all stages and incorporating a mix of strategies with measures for evaluation has been suggested when designing such initiatives [15].

**Fig. 3:**
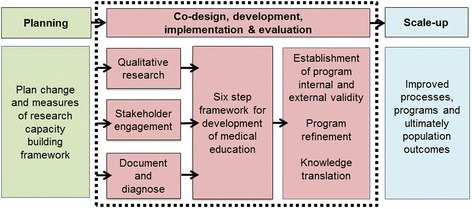
Program planning to scale up

Learning from the approaches described in the evidence, here we outline a capacity building program that involves clinical research and leadership education and support. The program is designed to facilitate engagement of health professionals to identify their needs and preferences and to enable them to drive clinically relevant research. In the absence of a co-ordinated approach to clinical research education and support in this large health service and Academic Health Sciences Centre, it will up-skill or enhance skills in clinical research methods. It will also build skills in leadership to engage, build and lead collaborative teams of multidisciplinary stakeholders through research and implementation of research findings in the clinical setting. Finally it will build skills in using research to guide practice as well as identify and address gaps in clinical practice. The success of the program will be measured in terms of health professional participant satisfaction and application of learned skills at the individual, team, organisational and external engagement levels according to research capacity building principles [14]. Whilst this program will be run locally in one of Australia’s largest health services, it will expand to one of the nation’s four accredited Academic Health Sciences Centres across multiple health services. We then aim to disseminate and scale up the approach to other health services if successful (Fig. 3).

In the short term, this project will contribute to the literature about effectiveness of education and support for health professionals as a strategy to increase health professional-led research to ensure that research is clinically relevant. It will provide information about: knowledge, attitudes and behaviours; and barriers, enablers and preferences of health professionals to use and conduct research; whether a capacity building program including education elements to provide and support skills in research methods, clinical research leadership and evidence-based practice is valued; and whether it influences engagement in clinical research. The findings from the project may be used to direct future clinical research engagement and capacity building research activity and funding. Furthermore, the findings from the project have the potential to guide future initiatives to engage health professionals in high quality research according to needs at the coal face of clinical care.

This clinical research engagement and leadership capacity building program is the first step in a longer term vision of the Monash Centre for Health Research and Implementation (MCHRI) and Monash Partners to address the recommendations of the McKeon review and embed a clinical research and healthcare improvement platform in the Monash Health system under the auspices of one of the largest Academic Health Sciences Centre in the world (Monash Partners). It is anticipated that research, led and conducted by health professionals, which addresses the clinical priority areas of the health setting, is more likely to be implemented and sustained in practice and lead to improved outcomes for both the health of the individual as well as the organisation and the community. The work described here will support research-enabled health professionals so that evidence gaps in the clinical setting are addressed by those who are providing the care.

### Ethics approval and consent to participate

Ethics approval, including all participant information and consent forms, survey and questionnaire, has been provided by the Monash Health Human Research Ethics Committee (Project 13313 L).

## Abbreviations

EBP: evidence-based practice
MCHRI: Monash Centre for Health Research and Implementation
